# Response of growing pigs to the inclusion of hybrid rye in low or high-energy diets

**DOI:** 10.1093/tas/txad137

**Published:** 2023-12-04

**Authors:** Miranda N Smit, Josiane C Panisson, A Denise Beaulieu

**Affiliations:** Prairie Swine Centre Inc., Saskatoon, S7H 5N9, SK, Canada; Prairie Swine Centre Inc., Saskatoon, S7H 5N9, SK, Canada; Department of Animal and Poultry Sciences, University of Saskatchewan, Saskatoon, S7N 5A8, SK, Canada

**Keywords:** dietary energy, digestibility, growth, rye, swine

## Abstract

Previous research has shown reduced feed intake and growth rate in pigs fed diets with hybrid rye replacing wheat. A reduction in growth rate caused by reduced feed intake will conceivably be counteracted by increasing the dietary energy level. Our objective, therefore, was to determine the effects of 40% hybrid rye inclusion replacing wheat in diets formulated to be either low or high net energy (NE) on growth, feed intake, energy digestibility, and lesion scores in growing-finishing pigs. We hypothesized that pigs fed 40% hybrid rye would perform better on the high than the low energy diets. A total of 160 pigs (body weight [BW] 70.1 kg) housed in 32 pens, 5 pigs per pen, were fed diets with 0% or 40% hybrid rye (var. ‘KWS Bono’; KWS LOCHOW GMBH), either with low (2,350) or high (2,450) kcal NE per kg of diet over two growth phases (phase 1; 70 to 85 kg BW; phase 2; 85 to 130 kg BW). The BW, and feed disappearance were measured on days 0, 8, 17, 28, 42, and 50. Fecal samples obtained in phase 2 (~100 kg BW) were used to calculate apparent total tract digestibility (ATTD) of gross energy (GE). Lesion scores were measured weekly. The ATTD of GE was unaffected by rye inclusion and was reduced in low vs. high NE diets. Overall, (days 0 to 50), pigs fed the low-energy rye diet gained 0.08 kg/d less (*P* < 0.01) than those fed the high-energy rye diet or the low-energy diet without rye, which was caused by a reduced weight gain during the initial 17 d of the trial. Final BW and overall feed intake were not affected by rye inclusion or NE level. The NE intake was greater (*P* < 0.05) and feed efficiency (G:F) was reduced (*P* < 0.05) in pigs fed rye diets compared to those fed diets without rye, whereas there was no effect of NE level on NE intake or G:F. There was no effect of rye inclusion or NE level on lesion scores. In conclusion, pigs can be fed diets including 40% hybrid rye with only minor changes in growth performance. Increasing the NE level of the first phase diet in the grower-finisher barn may be useful to avoid a reduction in growth performance when feeding hybrid rye.

## Teaser

The inclusion of 40% hybrid rye in diets for growing-finishing pigs resulted in only minor changes in growth performance. Increasing the energy level of the first phase diet in the grower-finisher barn may be useful to avoid a reduction in growth performance when feeding hybrid rye.

## Introduction

Access to a variety of grains and grain co-products allows the formulation of swine diets at the lowest cost. While it has been known for many years that rye can be used in swine feeds, the low acreages planted and susceptibility of rye to mycotoxins, specifically ergot, have limited its inclusion (Sullivan et al., 2005; Grela et al., 2023). However, development of new hybrid varieties with improved yields (Jürgens et al., 2012) and reduced susceptibility to ergot (Miedaner and Geiger, 2015) has renewed the interest in feeding rye to pigs. Net energy (NE; NRC, 2012) and standardized ileal digestible (SID) lysine content (Cervantes-Pahm et al., 2014) of rye are intermediate to those of wheat and barley. Hybrid rye also contains similar amounts of SID amino acids as corn and higher standardized total tract digestible P than corn (McGhee and Stein, 2018, 2019) and large amounts of highly digestible starch (McGhee and Stein, 2018, 2020), suggesting that rye has the potential to be a cost-effective ingredient in swine diets.

Recent research has demonstrated that the new hybrid rye varieties can replace wheat (Smit et al., 2019) or corn (McGhee et al., 2021; McGhee and Stein, 2023) in diets fed to growing pigs with no or minor effects on growth performance and carcass traits. Smit et al. (2019) observed reduced feed intake for pigs fed increasing hybrid rye replacing wheat, which in turn resulted in lower average daily weight gain. Typically, growing pigs will eat to meet their energy requirements (Whittemore et al., 2001), and therefore reduced feed intake maintains growth performance with high-energy diets (Smit et al., 2017, 2018). For example, pigs fed diets with energy ranging from 3,430 to 3,120 kcal digestible energy (DE)/kg had similar growth performance, but with decreased feed intake on the high-energy diets (Beaulieu et al., 2009). The current experiment was designed to determine if an increased dietary energy content will compensate for reduced feed intake of pigs fed diets formulated with hybrid rye replacing wheat.

Our objective was to determine the effects of 40% hybrid rye inclusion in diets formulated to be either low or high NE on growth, feed intake, energy digestibility, and lesion scores of growing pigs. We hypothesized that growing-finishing pigs fed 40% hybrid rye would perform better on the high than the low-energy diets.

## Materials and Methods

Study procedures were reviewed and animal use was approved by the University of Saskatchewan Animal Research Ethics Board and followed principles established by the Canadian Council on Animal Care (CCAC, 2009). The study was conducted at the Prairie Swine Center Inc. (Saskatoon, SK, Canada).

### Animals and Housing

A total of 160 pigs (PIC Camborough Plus females × C337 sires; PIC Canada Ltd., Winnipeg, MN, Canada) with an average initial body weight (BW) of 70.1 kg, were placed into two rooms, 16 pens per room, and five pigs per pen. Pens measured 2.4 m × 1.7 m. Flooring of each pen was fully slatted concrete, back, and sides of pens were concrete, with a front gate made of polyvinyl chloride planking. Each pen was equipped with a single-space dry feeder. A nipple drinker was located on the back wall of the pen. The room was ventilated using negative pressure and temperature was maintained within the thermo-neutral zone for pigs. Artificial light was provided for 12-h (0700 to 1900 h) followed by 12-h of darkness.

### Experimental Design and Diets

The trial utilized a randomized complete block design with a 2 × 2 factorial arrangement of treatments. Within an area block of four pens, barrows, or gilts were randomly allotted to diets with either 0% or 40% hybrid rye replacing wheat (Table 1), and with either low (2,350 kcal) or high (2,450 kcal) NE per kg of diet (Table 2), resulting in eight pens per treatment. Hybrid rye fed in this trial was the variety ‘KWS Bono’ developed by KWS LOCHOW GMBH (Bergen, Germany) and obtained from FP Genetics (Regina, SK, Canada). The low-energy diets were formulated to have similar dietary inclusion levels of all major ingredients compared to the high-energy diets, except for a decreased inclusion of canola oil.

**Table 1.** Analyzed nutrient content (%, as fed), ergot and mycotoxin content (ppb, as fed) of the rye (KWS Bono) used in the experimental diets

Nutrient, %		Mycotoxins, ppb (% of total ergot)	
Dry matter	88.2	Total ergot^1^	685
Crude protein	11.5	Ergocristin/inine	482 (70.4%)
Fat	1.3	Ergocryptin/inine	18 (2.6%)
Ash	1.7	Ergometrin/inine	44 (6.4%)
Acid detergent fiber	3.7	Ergosin/inine	35 (5.1%)
Neutral detergent fiber	13.7	Ergotamin/inine	102 (14.9%)
Calcium	0.06	Ergocornine/inine	4 (0.5%)
Phosphorus	0.28		
Arginine	0.59	Deoxynivalenol	106
Histidine	0.25	Aflatoxin B1	2.2
Isoleucine	0.31	α-Zearalenol	5.2
Leucine	0.72		
Lysine	0.44		
Methionine	0.34		
Methionine + cysteine	0.57		
Phenylalanine	0.53		
Threonine	0.30		
Tryptophan	0.16		
Valine	0.56		

^1^Combined *R* and *S* epimers are reported.

**Table 2.** Ingredient and nutrient composition (%, as fed) of the phase 1 (65 to 85 kg BW) and phase 2 (85 to 130 kg BW) diets formulated to contain 2,350 or 2,450 kcal NE/kg with or without 40% hybrid rye

	Phase 1^1^				Phase 2^1^^,^^2^			
Rye level, %	0		40		0		40	
NE level, kcal/kg	2,350	2,450	2,350	2,450	2,350	2,450	2,350	2,450
*Ingredients, %*								
Rye	0.0	0.0	40.0	40.0	0.0	0.0	40.0	40.0
Wheat	63.4	62.3	23.9	21.6	64.0	63.0	24.6	22.6
Barley	20.0	20.0	20.0	20.0	20.0	20.0	20.0	20.0
Canola meal	14.0	13.0	13.0	13.0	14.0	13.0	13.0	13.0
Canola oil	0.40	2.30	0.75	2.85	0.25	2.17	0.60	2.49
Vit/min premix^3^	0.20	0.20	0.20	0.20	0.20	0.20	0.20	0.20
Limestone	1.03	1.08	0.97	1.01	0.90	0.97	0.85	0.90
Dicalcium P	0.23	0.31	0.40	0.49	0.20	0.08	0.17	0.26
Salt	0.25	0.25	0.25	0.25	0.25	0.25	0.25	0.25
L-Lysine-HCl	0.35	0.46	0.41	0.44	0.21	0.26	0.22	0.26
DL-Methionine	0.00	0.00	0.03	0.03	0	0	0	0
L-Threonine	0.10	0.10	0.10	0.10	0.00	0.05	0.10	0.10
*Analyzed nutrients, %*								
Moisture	12.78	12.56	12.40	12.36	7.85	7.91	7.90	7.84
Starch	45.30	45.84	43.90	44.09	49.34	48.34	47.84	45.88
CP	15.44	14.81	14.45	14.50	16.49	16.20	15.44	15.32
Ether extract	2.12	3.52	2.56	3.96	1.79	2.09	1.82	2.69
Calcium	0.57	0.65	1.42	0.79	0.71	0.61	0.55	0.59
Phosphorus	0.52	0.49	0.48	0.50	0.54	0.48	0.47	0.49
Magnesium	0.19	0.18	0.18	0.18	0.20	0.18	0.18	0.18
Potassium	0.53	0.48	0.50	0.49	0.52	0.49	0.52	0.52
Sodium	0.10	0.11	0.17	0.13	0.35	0.20	0.16	0.15
ADF	5.36	4.53	4.80	4.66	6.32	5.59	5.80	5.64
NDF	12.84	11.06	10.99	11.80	13.27	12.17	13.27	14.46
TDF^4^^,^^5^	—	—	—	—	13.93	13.22	15.63	15.21
IDF^4^^,^^5^	—	—	—	—	12.54	11.79	13.28	13.06
SDF^4^^,^^5^	—	—	—	—	1.40	1.43	2.35	2.15
GE, kcal/kg^4^	—	—	—	—	3,781	3,917	3,881	3,964
*Nutrients as formulated*								
NE, Kcal/kg	2,343	2,444	2,343	2,445	2,343	2,444	2,343	2,443
Lysine, % SID	0.78	0.82	0.79	0.81	0.64	0.67	0.65	0.67
SID Lys/NE, mg/kcal	3.33	3.36	3.37	3.31	2.73	2.74	2.77	2.74
Ca, %	0.55	0.58	0.55	0.58	0.48	0.50	0.48	0.50
P, STTD	0.26	0.27	0.26	0.27	0.23	0.23	0.23	0.23
*Mycotoxins, ppm* ^6^								
Total ergot	0.03	<0.01	0.18	0.06	ND	ND	ND	ND
DON	0.08	0.10	0.08	0.17	ND	ND	ND	ND
*Feed cost, CAD* ^7^								
Per tonne	509.75	550.30	503.22	546.47	499.42	536.78	490.49	533.91

^1^Phase 1 was fed from days 0 to 17 and phase 2 from day 17 to market. The same diet was fed to barrows and gilts. Diets were formulated to meet requirements for gilts according to NRC 2012.

^2^Phase 2 diets fed for 1 wk prior and during fecal collections contained 0.4% celite as a source of acid-insoluble ash added to the diet as shown in Table 2. The analyzed nutrients shown in Table 2 are from the diet containing the celite.

^3^When added at the levels shown provided per kg of complete feed; 8,000 IU Vit A, 1,500 IU VitD, 30 IU Vit E, 2.0 mg menadione, 0.02 mg Vit B12, 1.0 mg thiamin, 0.10 mg biotin, 20 mg niacin, 4.0 mg riboflavin, 12 mg pantothenate, 0.50 mg folic acid, and 2.0 mg pyridoxine. Also provided per kg of complete feed; 100 mg Fe, 100 mg Zn, 40 mg Mn, 15 mg Cu, 0.3 mg Se, and 1.0 mg I.

^4^Only the phase 2 diets were analyzed.

^5^TDF, total dietary fiber; IDF, insoluble dietary fiber; SDF, soluble dietary fiber.

^6^Analyzed, ND, not detected.

^7^Average ingredient costs (in CAD) in Saskatchewan, Canada, between June and December 2022 were used in this analysis: Wheat grain $474, hybrid rye grain $433, barley grain $393, canola meal $491, canola oil $2,420, vit/min premix $14,593, limestone $145, dicalcium P $1,483, salt $70, L-Lysine-HCl $3,938, DL-Methionine $4,287, and L-Threonine $3,123.

Experimental diets were fed to slaughter weight over two growth phases (phase 1 [grower]; day 0 to 17; phase 2 [finisher]; and day 17—market). Diets had similar inclusion of barley and canola meal. Rye NE value was estimated based on previous research conducted at the Prairie Swine Center (unpublished) that provided a value of 3,509 kcal/kg for DE and chemical analysis of the hybrid rye was used to calculate the NE value using equation 1.8 of NRC (2012). Other values were from NRC (2012). A NE value of 2,472, 2,412, and 7,554 kcal/kg was used for wheat, rye, and canola oil respectively. Diets were formulated using gilt requirements, thus slightly overfeeding the barrows. Diets were formulated assuming caloric intake would be similar regardless of energy level, thus the SID Lys of the low-energy diets was reduced to keep the SID Lys/NE ratio constant (Table 2). Other amino acid ratios to Lys were according to the ideal protein concept (NRC, 2012). Premixes were added to meet or exceed vitamin and trace mineral requirements (NRC, 2012). A standardized total tract digestible phosphorus level of 0.22% and 0.15% was used for wheat and hybrid rye, respectively (NRC, 2012), resulting in greater inclusion of dicalcium phosphate in the diets containing rye. The first week of phase 2, 0.4% celite was added to the diets as a source of acid-insoluble ash (AIA). Pigs had free access to water and the assigned test diets were in pelleted form.

### Measurements and Calculations

Pigs were weighed at the initiation of feeding the experimental diets (day 0), and on days 8, 17, 28, 43, 50, and 57. Feed was weighed and delivered to pigs by hand and feed remaining in the pen feeder on weigh days was weighed back. Collected data were used to calculate pen average daily feed intake (ADFI), average daily weight gain (ADG), and feed efficiency expressed as ADG/ADFI (G:F).

Fecal samples were obtained by obtaining freshly excreted samples over a 24-h period, a minimum of one sample per pen, when pigs had received the phase 2 celite-containing diets for one week (~100 kg BW). Samples were only collected if defecation was observed and were immediately frozen (−20 °C). Apparent total tract digestibility (ATTD) of energy was calculated based on the indicator method using AIA as a marker and using the following equation 1 (Adeola, 2001):


$$\begin{array}{l} ATTD,\;\%=\left[1-(N_{F}\times M_{D})/(N_{D}\times M_{F})\right]\times 100\; \end{array}$$ (1)

Where *N*_*F*_ is the nutrient concentration in feces; *N*_*D*_ is the nutrient concentration in the diet; *M*_*D*_ is the marker concentration in the diet; and *M*_*F*_ is the marker concentration in feces.

NE of the diets was estimated according to NRC (2012) using the following equation 2:


$$\begin{array}{lll} NE=\; & (0.700\times DE)+(1.61\times EE)+(0.48\times Starch)\; & -(0.91\times CP)-(0.87\times ADF) \end{array}$$ (2)

Where DE is DE, EE is ether extract, crude protein (CP) is CP, and acid detergent fiber is acid detergent fiber.

Lesion scores were measured on the face, ear, body, and tail of each pig weekly in the morning and afternoon of the same day to distinguish between old lesions (morning score) and new lesions (afternoon minus morning score). The scores for the different body parts were summed to create one score per animal. The lesion scoring system, adapted from Roy et al. (2019), used the following scores: (0) no injury; (1) slight injury, <5 superficial wounds; (2) obvious injury, 5 to 10 superficial or < 3 deep wounds; (3) severe injury, >10 superficial or > 3 deep wounds.

### Chemical Analyses

Fecal samples were dried in a forced-air draft oven at 55 °C for about 72 h minimizing N loss (Jacobs et al., 2011). The feed, rye, and oven-dried feces were then ground through a 1-mm screen in a centrifugal mill (Model ZM 100, Retsch GmbH, Haan, Germany). The dry matter content of the feed and feces was measured by drying at 135 °C in an airflow-type oven for 2 h (method 930.15; AOAC, 2016). AIA content was analyzed in both feed and feces according to Van Keulen and Young (1977). The gross energy (GE) content of the feed and feces was analyzed using an adiabatic bomb calorimeter (6,400 automatic Isoperibol system, Parr Instruments Company, IL, USA) calibrated with benzoic acid. The total, insoluble, and soluble dietary fiber content of the feed was determined according to AOAC 991.43 (AOAC, 2016) using an ANKOM 200 fiber analyzer (ANKOM Technology, Macedon, NY, USA).

Rye samples were analyzed for moisture (AOAC 930.15), CP (AOAC 990.03[M]), ether extract (AOCS Am 5-04), ash (AOAC 923.03), acid detergent fiber (Ankom method 12[M]), neutral detergent fiber (Ankom method 13[M]), and amino acids (AA; AOAC 994.12) at the Central Testing Laboratories, Winnipeg, MB, Canada. Diets and rye samples were analyzed for mycotoxins and ergot alkaloids (EA) using UHPLC-MS/MS following solvent extraction at Prairie Diagnostic Services (Saskatoon, SK, Canada). A total of six EA were determined, *S* and *R* epimers combined.

### Statistical Analyses

Pen was the experimental unit for all variables. Because the sampling unit for BW and lesion scores was individual pigs, an average for each pen was calculated prior to statistical analyses.

Growth performance and energy digestibility were analyzed using the GLIMMIX procedure of SAS using a normal distribution. Models included the fixed effects of rye level (0% or 40%), energy level (low or high), sex (barrows or gilts), and interactions. Block was considered a random effect in the model. Overall ADG, ADFI, NE intake, and G:F were analyzed using closeout data. BW, ADG, ADFI, NE intake, and G:F were analyzed as repeated measures including growth phase as repeated term. Growth phase was added as a fixed effect and the interactions of growth phase with the other fixed effects were analyzed. An appropriate covariance structure was selected by comparing the goodness-of-fit measures of different structures. The Kenward-Roger correction was used for the denominator degrees of freedom. Since many pigs had already been sent for slaughter by day 57, growth performance data are only reported until day 50 on test.

Lesion scores were analyzed using the GLIMMIX procedure of SAS using a Poisson distribution and Log link function. The repeated measures analysis for lesion scores was overparameterized, so the model was simplified. The lesion score data for both old and new lesions was analyzed for each week separately.

To test the hypotheses, *P* < 0.05 was considered significant and *P* < 0.10 was a trend.

## Results

### Dietary Nutrients and Energy Digestibility

The hybrid rye fed in this experiment had relatively low levels of mycotoxins, including EA. Ergocristine (70% of total measured EA) was the most common EA measured, followed by ergotamine (15%, Table 1). Including hybrid rye replacing wheat generally decreased dietary starch and CP content, whereas content of crude fat and the different fiber fractions (neutral detergent fiber, TDF, IDF, and SDF) increased. As designed, dietary nutrient composition was very similar between the high and low-energy diets, except for lower crude fat content of the low-energy diets (Table 2).

Energy digestibility and the DE content of the diet were reduced (*P* ≤ 0.001) in the low relative to the high-energy diets. The ATTD of GE and the DE content on a dry matter basis were unaffected by rye inclusion; however, DE content on an as fed basis was greater (*P* < 0.05) in the 40% rye diets compared to the 0% rye diets (Table 3). Calculated dietary NE values were close to formulated values, although the low-energy diet without rye had a slightly lower NE value than expected (Table 3).

**Table 3.** Apparent total tract digestibility (ATTD) and digestible energy (DE) value of the phase 2 (85 to 130 kg BW) grower-finisher diets with or without 40% hybrid rye^1^ inclusion^2^ and low or high in NE

	Rye		NE		0% Rye Low NE	0% Rye High NE	40% Rye Low NE	40% Rye High NE	Pooled SEM	*P-*values		
	0%	40%	Low	High						Rye	NE	Rye*NE
ATTD of GE, %	80.5	80.5	79.4	81.6	79.4	81.7	79.5	81.5	0.52	0.979	0.001	0.783
DE, DM basis, kcal/kg	3,561	3,586	3,482	3,664	3,453	3,669	3,512	3,660	23.0	0.295	<0.001	0.158
DE, as fed, kcal/kg	3,099	3,158	3,043	3,214	3,001	3,198	3,085	3,230	20.2	0.013	<0.001	0.225
NE, as fed, kcal/kg^3^					2,288	2,435	2,333	2,436				

^1^KWS LOCHOW GMBH (Bergen, Germany).

^2^LSmeans based on eight pens of five pigs each per hybrid rye level × NE level.

^3^Calculated with equation 1 to 8 from NRC (2012) and using DE as fed values from this Table.

### Growth Performance

Two pigs were removed from the experiment for reasons unrelated to treatment.

There were no three-way interactions among hybrid rye inclusion, NE level, and sex for growth performance parameters. Main effects of sex were as expected and are not presented. Except for those described below, there were no interactions between hybrid rye inclusion and NE content.

Overall, (days 0 to 50), there was an interaction (*P* < 0.01) between hybrid rye inclusion and NE level for ADG; pigs fed the low-energy rye diet gained 0.08 kg/d less than those fed the high-energy rye diet or the low-energy diet without rye, with those fed the high energy diet without rye being intermediate (Table 4). For the entire trial, ADFI was not affected by rye inclusion or NE level. For the entire trial, NE intake was greater (*P* < 0.05) and feed efficiency (G:F) was reduced (*P* < 0.05) in pigs fed rye diets compared to those fed diets without rye, whereas there was no effect of NE level on NE intake or feed efficiency (Table 4).

**Table 4.** Growth performance per growth phase and overall (days 0 to 50) of grower-finisher pigs fed high or low NE diets with or without 40% hybrid rye^1^ inclusion^2^

	Rye		NE		0% Rye Low NE	0% Rye High NE	40% Rye Low NE	40% Rye High NE	Pooled SEM	*P-*values		
	0%	40%	Low	High						Rye	NE	Rye*NE
BW, kg/d												
Day 0	70.1	70.2	70.2	70.1	70.1	70.1	70.2	70.1	0.38	0.791	0.749	0.981
Day 8	80.4	78.8	78.9	80.3	80.4^z^	80.5^z^	77.5^y^	80.1^z^	0.46	<0.001	<0.001	<0.001
Day 17	92.9	90.3	90.7	92.5	92.9^z^	92.9^z^	88.6^y^	92.1^z^	0.53	<0.001	<0.001	<0.001
Day 28	106.8	105.4	105.6	106.6	107.1^z^	106.4^z^	104.1^y^	106.7^z^	0.69	0.008	0.058	<0.001
Day 43	123.2	122.0	122.5	122.7	124.0	122.4	121.1	122.9	1.23	0.171	0.897	0.144
Day 50	128.4	126.7	127.4	127.7	128.9	127.9	125.9	127.6	1.47	0.125	0.732	0.252
ADG, kg/d												
Overall (days 0 to 50)	1.18	1.16	1.16	1.18	1.20^z^	1.17^z,y^	1.12^y^	1.20^z^	0.018	0.234	0.231	0.007
Days 0 to 8	1.29	1.08	1.09	1.27	1.28^z^	1.30^z^	0.90^y^	1.25^z^	0.029	<0.001	<0.001	<0.001
Days 8 to 17	1.38	1.29	1.31	1.36	1.39^z^	1.38^z^	1.23^y^	1.34^z^	0.027	0.002	0.092	0.002
Days 17 to 28	1.26	1.37	1.35	1.28	1.30^y^	1.23^x^	1.41^z^	1.33^y^	0.022	<0.001	0.003	<0.001
Days 28 to 43	1.08	1.13	1.11	1.10	1.10	1.07	1.12	1.13	0.039	0.234	0.760	0.604
Days 43 to 50	0.85	0.79	0.81	0.83	0.88	0.83	0.75	0.83	0.046	0.178	0.706	0.310
ADFI, kg/d												
Overall (days 0 to 50)	3.43	3.47	3.49	3.41	3.50	3.36	3.47	3.46	0.057	0.502	0.135	0.190
Days 0 to 8	2.88	2.75	2.78	2.85	2.87	2.88	2.69	2.81	0.044	0.008	0.123	na^3^
Days 8 to 17	3.87	3.82	3.85	3.85	3.89	3.86	3.81	3.83	0.148	0.720	0.993	na
Days 17 to 28	3.63	3.65	3.71	3.57	3.72	3.53	3.70	3.61	0.042	0.550	0.003	na
Days 28 to 43	3.42	3.50	3.53	3.40	3.53	3.31	3.53	3.48	0.072	0.261	0.082	na
Days 43 to 50	3.20	3.53	3.41	3.32	3.31	3.09	3.50	3.57	0.221	0.142	0.717	na
NE intake, kcal/d												
Overall (days 17 to 50)	8,117	8,484	8,229	8,373	8,114	8,121	8,344	8,624	161	0.032	0.382	0.406
Days 17 to 28	8,553	8,703	8,569	8,688	8,516	8,591	8,621	8,785	101	na	na	na
Days 28 to 43	8,066	8,350	8,147	8,269	8,068	8,065	8,226	8,474	173	na	na	na
Days 43 to 50	7,542	8,427	7,871	8,098	7,579	7,504	8,162	8,693	528	na	na	na
Feed efficiency (G:F, kg/kg)												
Overall (days 0 to 50)	0.35	0.33	0.33	0.34	0.34	0.35	0.33	0.34	0.010	0.028	0.222	0.652
Days 0 to 8	0.45	0.39	0.39	0.45	0.45^z^	0.45^z^	0.34^y^	0.44^z^	0.01	<0.001	<0.001	<0.001
Days 8 to 17	0.36	0.34	0.34	0.36	0.36	0.36	0.33	0.35	0.013	0.118	0.262	0.186
Days 17 to 28	0.35	0.38	0.37	0.36	0.35^y^	0.35^y^	0.38^z^	0.37^z^	0.005	<0.001	0.222	<0.001
Days 28 to 43	0.32	0.32	0.31	0.33	0.31	0.32	0.32	0.33	0.006	0.305	0.098	0.293
Days 43 to 50	0.27	0.23	0.24	0.26	0.27^z^	0.27^z^	0.22^y^	0.24^z,y^	0.016	0.019	0.377	0.084

^x,y,z^Means within a row without a common superscript differ (*P* < 0.050).

^1^KWS LOCHOW GMBH (Bergen, Germany).

^2^LSmeans based on eight pens of five pigs each per hybrid rye level × NE level.

^3^na, not applicable. *P*-values for each period are only given when there was an interaction between the parameter and time in the repeated measures analysis.

An examination of individual growth phases revealed an interaction (*P* < 0.001) between hybrid rye inclusion and NE level for BW at days 8, 17, and 28. Pigs fed the low-energy rye diets weighed less than those fed high-energy rye diets or diets without rye (Table 4). BW was not significantly affected by rye inclusion or NE level on days 43 and 50 (Table 4).

There was an interaction (*P* < 0.01) between hybrid rye inclusion and NE level for ADG for the days 0 to 8 and days 8 to 17 periods; pigs fed the low-energy rye diets gained less than those fed the high energy rye diet or the diets without rye. For the days 17 to 28 period, the interaction (*P* < 0.001) between rye inclusion and NE level for ADG was reversed; pigs fed the low-energy rye diet gained the most weight, and those fed the high-energy diet without rye gained the least, with the other two treatments being intermediate (Table 4). There was no effect of rye inclusion or NE level on ADG for the days 28 to 43 and days 43 to 50 periods.

For the days 0 to 8 period, rye inclusion resulted in decreased ADFI by 0.13 kg/d (*P* < 0.01), whereas ADFI was not affected by rye inclusion in any of the other growth phases. Feed intake was 0.14 kg/d greater (*P* < 0.01) during the days 17 to 28 period and tended (*P* = 0.082) to be 0.13 kg/d greater during the days 28 to 43 period in pigs fed low relative to high NE diets. Energy level did not affect ADFI for the days 0 to 8, 8 to 17, and 43 to 50 periods (Table 4).

NE intake was consistently greater for pigs fed diets with rye compared to those fed diets without rye in all measured growth periods (days 17 to 28, 28 to 43, and 43 to 50). There was no effect of NE level on NE intake throughout the trial (Table 4).

For the days 0 to 8 period, feed efficiency (G:F) was reduced (*P* < 0.001) in pigs fed the low-energy rye diet compared to pigs fed the high-energy rye diet or diets without rye. For the days 17 to 28 period, pigs fed rye diets had better G:F than those fed diets without rye (*P* < 0.001), whereas, for the days 43 to 50 period, pigs fed the low-energy rye diet tended to have lower feed efficiency compared to pigs fed the wheat diets, while the high energy rye diet was intermediate (*P* = 0.084). For the days 28 to 43 period, pigs fed low NE diets tended to have worse G:F compared to pigs fed high NE diets (Table 4).

### Lesion Scores

Minimal aggression occurred throughout the trial, resulting in low lesion scores for old lesions (average of 1.12, 1.15, 1.01, 0.90, and 1.07 for weeks 2 to 6, respectively) or new lesions (average of 0.37, 0.15, 0.17, 0.17, and 0.08 for weeks 2 to 6, respectively). Overall average lesion score for old lesions was 1.05 both for pigs fed diets with or without rye, and 1.04 and 1.06 for pigs fed diets low or high in energy, respectively. Overall average lesion score for new lesions was 0.21 and 0.17 for pigs fed diets without or with rye, respectively, and was 0.23 and 0.16 for pigs fed diets low or high in energy, respectively. There was no effect of rye inclusion (*P* > 0.38) or NE level (*P* > 0.37) on lesion scores for old and new lesions throughout the experiment (data not shown).

## Discussion

### Diet Formulation, Dietary Nutrients, and Energy Digestibility

The DE and ME systems ignore energy losses due to heat increment, meaning that they overestimate the energy available to support maintenance and growth for ingredients high in fiber or non-starch polysaccharides (NSP; Zijlstra and Beltranena, 2013). Therefore, the NE system is particularly useful when feeding ingredients high in fiber or NSP. In this trial, the hybrid rye-containing diets were formulated to have similar NE levels as the wheat control diets. Similar to Smit et al. (2019) we added canola oil to hybrid rye-containing diets to compensate for the lower NE content of rye relative to wheat. The result of this is a potential confounding effect between hybrid rye and canola oil in our trial. Moreover, the NE level of canola oil is an approximate value, meaning that it is difficult to keep the NE level of diets constant when adding canola oil. The alternative method would be to keep the oil level and all other major ingredients the same when replacing wheat or corn with hybrid rye, resulting in decreased energy content of the hybrid rye diets. This was the approach taken by McGhee et al. (2021) and McGhee and Stein (2023) when they compared hybrid rye to corn diets, resulting in decreasing ME levels for their diets with increasing hybrid rye inclusion. Dietary energy level affects feed intake (Beaulieu et al., 2009; Smit et al., 2017, 2018, 2021), meaning that in these trials there was a confounding effect between hybrid rye and dietary energy level. In our trial, we were specifically interested in increasing the NE level of the hybrid rye diet to potentially overcome the previously observed reduction in feed intake (Smit et al., 2019), and therefore, it was important to keep the NE level of the hybrid rye diets the same as the wheat diets.

The hybrid rye fed in this trial had low levels of mycotoxins, including EA. Traditionally, rye was not commonly fed to pigs due to concerns over high EA contamination that could reduce feed intake and affect growth performance (Friend and MacIntyre, 1970). Indeed, analysis of grain samples from western Canada across 25 years showed 5- to 10-fold greater ergot incidence and 4 times greater ergot severity occurred in rye vs.. wheat (Walkowiak et al., 2022). The new hybrid rye varieties have been bred to be more resistant to EA infection due to the production of vast amounts of pollen that overwhelm the stigma giving mold spores a lower chance of infecting the ear before the stigma closes (Miedaner and Geiger, 2015). Our analysis showed that EA were still present in hybrid rye, but our value of 0.685 mg/kg was much lower than the 1.14 mg/kg EA observed in a traditional rye sample from Canada (Tittlemier et al., 2015). The hybrid rye-containing diets had greater fat content than the wheat control diets, likely due to the higher canola oil level in the hybrid rye diets. The hybrid rye diet also had greater fiber content due to rye having greater fiber content than wheat (McGhee and Stein, 2020).

ATTD of GE in the hybrid rye diets was similar to the wheat diets, despite the fact that hybrid rye has a slightly lower ATTD of GE than wheat (~85% vs. 87.4%, respectively; McGhee and Stein, 2020). It is possible that the greater amount of canola oil in the hybrid rye diets played a role, because ATTD of GE in oil (~88 - 90%; Li et al., 2018) is greater than in wheat or rye (~87 and 85%, respectively; McGhee and Stein, 2020) and the rye diets contained more canola oil than the control diets. Moreover, dietary inclusion of canola oil may increase digestibility of CP and energy originating from the other ingredients of the diet (Zhou et al., 2017). A slightly greater DE value in the hybrid rye relative to the wheat diets was expected, because the diets were formulated to have equal NE levels. As hybrid rye has a greater fiber and NSP content than wheat (Smit et al., 2019; McGhee and Stein, 2020), energy losses due to heat increment will be bigger in rye than wheat, meaning that the DE level of the diet would need to be slightly greater to provide the same level of NE.

As expected, the high NE diets had a greater DE value than the low NE diets. The high NE diets had greater oil content compared with the low NE diets due to the greater canola oil content, likely increasing the ATTD of GE of the high vs. the low NE diets. Fiber content was slightly lower for high relative to the low NE diets, which may have also played a role in the improved ATTD of GE for high vs. low NE diets. The NE value of the low-energy diets averaged 2,310 kcal/kg compared to 2,440 kcal/kg for the high-energy diets. Grower-finisher pigs are often fed diets above 2,350 kcal/kg, but it is not uncommon for lower NE values to be used, especially when alternative feed ingredients are used (Beltranena and Smit, 2015). Previous research has shown that pigs can be fed dietary NE levels as low as 2,100 kcal/kg (Smit et al., 2017) without notable changes in growth rate.

### Growth Performance

Several trials have shown a slight reduction in feed intake when hybrid rye replaces corn (McGhee et al, 2021; McGhee and Stein, 2023) or wheat (Smit et al., 2019) in grower-finisher diets. The protocol utilized by McGhee et al. (2021) and McGhee and Stein (2023) allowed the ME level of the diets to fall when hybrid rye replaced different amounts of corn in the diets. A decrease in feed intake in the hybrid rye diets was therefore surprising, because pigs generally compensate for lower dietary energy levels by increasing feed intake, both when differences in energy level are realized through changes in oil/fat addition (Smith et al., 1999; De La Llata et al., 2001) or through changes in basal ingredients (Beaulieu et al., 2009; Smit et al., 2017).

In our trial, we aimed to maintain the caloric intake of pigs fed diets with 40% hybrid rye by increasing the NE level of the diets from 2,350 to 2,450 kcal/kg. In the first 8 d of the trial, feed intake was lower in pigs fed the hybrid rye-containing diets than the wheat control diets. Pigs were fed wheat-based diets before the start of the trial, so it is possible that they needed the first 8 d of the trial to adapt to hybrid rye. McGhee et al. (2021) also showed reduced feed intake in the first but not the second phase of their trial, which they also suggested may have been due to an adaptation effect. The decreased feed intake may have also partially been due to the increased dietary fiber content in rye, which may increase gut fill and water binding capacity in the gastrointestinal tract, thereby limiting the ability of younger pigs to consume more feed (Ndou et al., 2013). In contrast to our trial, Sullivan et al. (2022) and Smit et al. (2019) noticed a reduction in overall feed intake with increasing hybrid rye inclusion, which in the case of Smit et al. (2019) was due to reduced feed intake in the finisher but not the grower phase. In both cases, hybrid rye replaced a proportion of corn (Sullivan et al., 2022) or wheat (Smit et al., 2019), rather than having a fixed hybrid rye inclusion level as was the case in our trial. Because the proportion of grain in the diet increases as pigs grow older, this means that in both trials the hybrid rye inclusion level increased as pigs aged (from maximum of 45% in grower 2 to maximum of 66% in the finisher 2 phase for Smit et al. [2019]). In contrast, hybrid rye inclusion level was kept constant at 40% throughout the current trial. In our trial, there was an increased caloric (NE) intake for days 17 to 50 for pigs fed the hybrid rye vs. the wheat diets despite a lack of effect on feed intake during this time. This was likely due to a numerical increase in feed intake between days 17 and 50 when pigs were fed hybrid rye, combined with a slightly greater NE level for the rye vs. wheat diets.

In the first 8 d, the interaction between hybrid rye inclusion and NE level in weight gain was coupled with an interaction in feed efficiency, showing the lowest weight gain and feed efficiency for pigs fed the low NE hybrid rye diets. From days 8 to 17, the interaction between hybrid rye inclusion and NE level in weight gain was more surprising, because there was no effect of hybrid rye or NE level and no interaction between the two on feed intake nor feed efficiency; however, numerically pigs on the low NE hybrid rye diets had the lowest feed intake and feed efficiency, resulting in a significantly lower weight gain. Providing a higher NE level helped pigs on the hybrid rye diets to overcome the lower growth rate due to the rye during this time. Interestingly, on days 17 to 28, the interaction in weight gain was reversed, with pigs fed the low NE hybrid rye diets showing the greatest weight gain and best feed efficiency of all diets, perhaps indicative of compensatory growth. These data show that in younger animals, or with the initial introduction of rye, the NE level of the diet may need to be considered when formulating diets including hybrid rye.

It was surprising that there was no effect of NE level on feed intake in the first 17 d. Initial BW in this trial was ~70 kg, and the pigs should have the gut capacity to increase feed intake to compensate for the lower dietary energy level (Whittemore et al., 2001). Indeed, this was observed in our previous trials (Smit et al., 2017, 2021). Moreover, ADG was not affected by NE level in the wheat control diets in the first 17 d, even though caloric intake must have been lower for pigs fed the low vs. high NE diets. From day 17 onwards, pigs on the low NE diets were able to increase their feed intake enough to maintain caloric intake. Despite the similar NE intake, ADG was improved in pigs fed the low vs. high NE control diets for days 17 to 28. These results suggest a greater caloric efficiency from days 0 to 28 when feeding low vs. high NE diets. This is in agreement with our previous results (Smit et al., 2017, 2018, 2021). Considering the lack of effect of NE level on ADG between days 28 and 50 and similar NE intake, caloric efficiency was probably similar for pigs fed low and high NE diets during this time.

### Lesion Scores

When pigs fight, they target the head, neck, and ears of their opponent using bites and slashes from the canine teeth, resulting in the accumulation of superficial skin lesions (McGlone, 1985). Skin lesions are caused by both engagement in reciprocal fighting and being bullied. The total number of skin lesions can be used as an indicator of aggression in grower-finisher pigs (Turner et al., 2006). We hypothesized that pigs fed hybrid rye would show reduced aggression, resulting in reduced lesion scores, due to the increased fiber and NSP content in rye causing a higher extract viscosity, water holding capacity, and swelling capacity (Abd El-Wahab et al., 2021), as well as delayed stomach emptying compared to wheat (Wilke et al., 2021), all of which may result in a longer feeling of satiety. However, if pigs would feel satiated longer, the feed intake would be expected to be reduced as a result. Lesion scoring did not start until the second week of the trial until week 6. During this time, feed intake was not affected by rye inclusion, so it is not surprising that lesion scores were also not affected by rye inclusion.

We also hypothesized that pigs fed low NE diets would show more aggression at the feeder compared with pigs fed high NE diets, due to their need to eat more to make up for the lower energy density of the feed. However, dietary NE level did not significantly affect lesion scores. This may be due to the fact that only five pigs had to share a feeder. In a previous trial, Smit et al. (2021) did not find interactions between dietary NE level, group size, and feeder space. They used pens of 22 pigs with two feeder spaces and pigs were fed diets with a NE level of 2,200 kcal. Those pigs were able to increase feed intake enough to have similar growth rates to pigs fed 2,350 kcal NE diets. Adding a feeder space increased feed disappearance without affecting growth rate, suggesting that more feed was wasted rather than pigs eating more. Having only five pigs in a pen with one feeder likely meant pigs were able to spend more time at the feeder to increase feed intake without being in each other’s way. Indeed, between 12 (Gonyou and Lou, 2000) and 20 (Spoolder et al., 1999) pigs can feed on a feeder space without affecting productivity.

Aggression was very low in general during this trial, which may again be due to the small group size per pen, as well as the provision of sufficient feeder space and floor space to the pigs. Pigs were also not mixed after their initial mixing before the start of the experiment, so a hierarchy was established before the start of the trial and was likely maintained throughout the trial. It is possible that an effect of hybrid rye inclusion and/or dietary NE level on lesion scores may be seen in barns that have higher levels of aggression in their grow-finish facilities. Research into these effects in a commercial barn would be of interest.

Eight pens per treatment may not be sufficient to detect differences in lesion scores. For example, lesion scores were almost doubled in barrows vs. gilts (data not shown), yet the analysis did not find a significant effect of sex. For this reason, a larger sample size may need to be considered in future trials.

### Feed Cost

Due to its lower NE value compared to wheat, when hybrid rye is replacing wheat in the diet the oil inclusion level needs to be increased. As a result, the price of not only wheat and hybrid rye but also oil needs to be considered when determining whether to formulate diets with hybrid rye. Smit et al. (2019) showed no effect of hybrid rye inclusion level on feed cost per pig, nor on feed cost per kg BW gain due to a lack of effect on feed efficiency in that trial. For the current trial, feed cost calculations (data not shown) showed no difference in feed cost per pig between the hybrid rye vs. wheat control diets but feed cost per kg BW gain tended to be a bit higher for pigs fed the hybrid rye diets, due to the lower feed efficiency of the hybrid rye diets throughout the trial.

The low NE diets were much cheaper than the high NE diets. This large difference in feed cost per tonne translated to lower feed cost per pig and per kg BW gain for pigs fed low vs. high NE diets. This finding is in agreement with our previous research that has consistently shown lower feed cost per pig and per kg BW gain, as well as higher income subtracting feed cost when pigs are fed lower energy diets (Beaulieu et al., 2009; Beltranena and Smit, 2015; Smit et al., 2021).

## Conclusion

In conclusion, feeding 40% hybrid rye replacing wheat to grower-finisher pigs resulted in similar overall feed intake and final BW, slightly lower overall feed efficiency, and lower overall growth rate for pigs fed the low NE but not the high NE rye diets. The lower overall growth rate was caused by reduced BW gain during the initial 17 d of the trial and it may be useful to consider increasing the NE level of the first phase diet in the grower-finisher phase when including hybrid rye to avoid this reduction in growth performance.
